# miR-195-5p regulates cell proliferation, apoptosis, and invasion of thyroid cancer by targeting telomerase reverse transcriptase

**DOI:** 10.1080/21655979.2021.1963908

**Published:** 2021-09-05

**Authors:** Zhiwen Liu, Li Zhang, Wen Chen, Fenqian Yuan, Zhi Yang, Sheng Liu, Fei Le

**Affiliations:** a Department Of Neonatal Surgery, Jiangxi Provincial Children's Hospital, Nanchang, Jiangxi, China; b Electrocardiography Room, Jiangxi Provincial Cancer Hospital, Jiangxi Cancer Hospital of Nanchang University ,Jiangxi Provincial Key Laboratory of Translational Medicine and Oncology ,Jiangxi Cancer Center, Nanchang, Jiangxi, China; c Department Of Breast Surgery, Jiangxi Provincial Cancer Hospital, Jiangxi Cancer Hospital of Nanchang University, Jiangxi Provincial Key Laboratory of Translational Medicine and Oncology, Jiangxi Cancer Center, Nanchang, Jiangxi, China; d Department Of Head And Neck Surgery, Jiangxi Provincial Cancer Hospital, Jiangxi Cancer Hospital of Nanchang University, Jiangxi Provincial Key Laboratory of Translational Medicine and Oncology, Jiangxi Cancer Center, Nanchang, Jiangxi, China; e Department Of Thoracic Surgery, The First Affiliated Hospital of Nanchang University, Nanchang, Jiangxi, China

**Keywords:** MicroRNA-195-5p, telomerase reverse transcriptase, cell development, human thyroid cancer

## Abstract

In most human primary cancers, the expression, or telomerase activity, of telomerase reverse transcriptase (*TERT*) is detectable. However, the mechanism of*TERT*activity within oncogenesis of thyroid cancer remains largely unknown. In this study, we identified miR-195-5p as having involvement in cell proliferation, apoptosis, and invasion in human thyroid cancer. MTT was used to measure cell proliferation, Transwell chamber was used to measure invasion. Western blotting was used to detect the expressions of TERT, PCNA, and Ki67. Target gene prediction software predicted that TERT may be the target gene of miR-195-5p. Luciferase reporting system was used to identify the targeting relationship. A significant increase of in TERT expression was observed by immunohistochemistry compared with normal tissue, however, a decrease in miR-195-5p expression using qRT-PCRand western blot compared with normal cells. Functional analysis demonstrates that miR-195-5p negatively correlated with*TERT*and inhibited*TERT*expression through its interaction with the*TERT*3ʹ-untranslatedregion (3ʹ-UTR). Overexpression of miR-195-5p was shown to inhibit proliferation and invasion, and promote apoptosis of CAL-62 thyroid cancer cells. miR-195-5p-mediatedeffects were rescued by the overexpression of*TERT*. Altogether, our data demonstrate that miR-195-5p regulates cell proliferation, apoptosis, and invasion in human thyroid cancer via*TERT*, providing evidence of a new potential therapeutic target for further investigation.

## Introduction

Thyroid cancer is a common tumor of the endocrine system, with increasing incidence rates and a tendency for younger aged adults having an onset of the disease [1,2]. There are four main types of thyroid cancer according to its pathological changes: Papillary Thyroid Carcinoma (PTC), Follicular Thyroid Carcinoma, Medullary Thyroid Carcinoma, and undifferentiated Thyroid cancer (Anaplastic Thyroid Carcinoma, ATC) [3–6]. Clinical surgery is the main treatment option as well as endocrine therapy, chemotherapy, gene therapy, and other methods for thyroid cancer [7,8]. With the analysis of the characteristics of differentiated thyroid cancer and combining with the physiological characteristics of postmenopausal women, this study evaluates the factors influencing endocrine therapy post thyroid cancer operation, develops a reasonable endocrine therapy program, and instructs the clinical patients in thyrotropin (TSH) inhibition therapy [9,10]. Xia et al have previously studied the sensitivity of thyroid cancer to chemotherapy [11]. They were able to demonstrate that patients who had undergone thyroid cancer resection routinely needed to take thyroid hormone medication for their lifetime. Therefore, gene therapy has become a more favorable and potential treatment method.

In recent years, a large number of studies have shown that long non-codingRNAs (lncR-NAs)and microRNA (miRNA) participate in the occurrence and development of thyroid cancer [12,13]. TERT is a ribonucleoprotein polymerase that maintains telomere ends by adding the telomere repeat sequence TTAGG, because it is usually suppressed in somatic cells after birth, telomeres gradually shorten. The regulation and relaxation of TERT expression in somatic cells may be related to tumorigenesis [14,15]. The overexpression of Telomerase reverse transcriptase (TERT) occurs in various malignant tumors [16]. In contrast, miR-195-5p is downregulated in various malignant tumors such as breast, colorectal, and gastric cancers, and is associated with tumor cell proliferation, apoptosis and invasion, and migration [17]. It has been reported that TERT can participate in tumor development through downregulation of miR-195-5p [18]. However, there are few reports on the expression and correlation of TERT and miR-195-5p in PTC patients.

The present study aims to evaluate the regulatory effect of overexpression of miR-195-5p on the proliferation, apoptosis, and invasion of thyroid cancer cells, CAL-62, to understand the effect that overexpression of miR-195-5p has on the proliferation of them. The mechanism of apoptosis and invasion provides a certain theoretical basis for the treatment of thyroid cancer. In this study, we conducted high-volumesequencing on thyroid tissues from thyroid cancer patients and non-canceroussamples, with differential gene expression between the two groups was noted. Among them, miRNA-195-5p and TERT were differentially expressed. Therefore, this study focused on the relationship between miRNA-195-5p and TERT in thyroid cancer.

## Materials and methods

### Clinical tumor specimens

In this study, 120 patients admitted to Jiangxi Provincial Cancer Hospital between June 2019 and December 2020 were included. All underwent surgical resection with thyroid lesions, and the tissue specimens were removed from the incised end (tumor edge >5 cm), thyroid carcinoma was confirmed by pathology, and normal tissue samples as reference tissue adjacent to carcinoma were obtained. No patients included in this study had a history of malignancy, had not had prior radiotherapy treatment, chemotherapy, or immune therapy. The patients were not distinguished between age, gender, medical history, tumor grade, and other factors. All patients provided informed consent and the study was approved by the Research Ethics Committee of Jiangxi Provincial Cancer Hospital.

### Cell culture and transfection

The American Type Culture Collection (ATCC, USA) provided non-cancerousthyroid cells, HTori-3, and thyroid cancer cells CAL-62. The thyroid cancer cell line CAL-62 was cultured in Dulbecco’s modified eagle’s medium (Sigma-Aldrich,USA) with 1% penicillin/streptomycin and 10% fetal bovine serum (Sigma-Aldrich,USA). They subsequently underwent transfection of oligonucleotides (50 nm, miR-195-5p mimics, NC-mimics,miR-195-5p inhibitor, NC-inhibitor,and si-TERT,respectively), and Lipofectamine 2000 (Invitrogen, Carlsbad, CA) was used according to the manufacturer’s instructions.

### Apoptosis by flow cytometry

Collect cells(1 × 10^6^cells/mL) 48 h after irradiation, centrifuge at 1000 r/min for 5 min, then discard the medium, wash once with PBS, discard PBS; add 250*μ*L PBS to resuspend the cells, and finally add 10*μ*L annexin V-FITC and 5*μ*L PI, mix well, incubate at room temperature in the dark for 15 min, and detect by flow cytometry.

### MTT assay

A total of1000–10000 cells per well were seeded into 96-wellplates with a volume of 200ul of media containing 10% fetal bovine serum per well. |Cells were cultured for 3–5 days, where 20ul of MTT solution(5 mg/ mL prepared with PBS, pH = 7.4) was added to each well, and further incubated for 4 h. After the incubation period, the culture supernatant was discarded from each well, for suspension cells, were centrifuged for pellet collection to the cells, 150 ul of DMSO was added to each well, oscillated for 10 min, ensuring all formazan crystals fully dissolved, then culture plates were read at 490 nm wavelength.

### Quantitativereal-time polymerase chain reaction

Total RNA from lung cancer tissue samples were extracted using Trizol at 4°C (Invitrogen), and the isolated RNA was reverse transcribed into cDNA according to manufacturer’s instructions (Takara). ABI PRISM7900 Sequence Detection System (Applied Biosystems, USA) with PowerUp™ SYBR® Green Master Mix (Thermo Fisher Scientific, USA) was used to perform quantitative RT-PCR.The2^−ΔΔCt^method was used for the analysis of the relative expression levels of mRNA and miRNA, and U6 served as an internal control [19].

### Luciferase reporter assay

An online tool, TargetScan (http://www.targetscan.org/vert_72/) was used to predict the potential targets of the miRNAs. The mutant (MUT) TERT-3ʹ-UTRand wild-type(WT) TERT-3ʹ-UTRcontaining the miR-195-5p putative binding site were synthesized and inserted into the pmirGLO dual-luciferasereporter vectors (YouBio, Changsha, China). The reporter vectors containing the MUT or WT of TERT 3ʹ-UTRand NC-mimics/miR-195-5p mimics were co-transfectedinto HEK293T cells, which cultured at 45% confluence. After 48 h, the dual-luciferaseassay system (Promega, Madison, USA) was used to measure the luciferase activities, normalized to Renilla luciferase.

### Western blotting

TERT, proliferating nuclear antigen 67 (Ki67), proliferating cell nuclear antigen (PCNA), Caspase 3 (Caspase-3), and Caspase 9 (Caspase-9) antibodies were all used at 1:1000 dilution (Shanghai Anyan Co., Ltd., Shanghai, China). The optical density value of the protein of interest vs. GAPDH control protein optical density value was used to calculate the relative protein expression levels.

### Invasion by transwell

Matrigel was diluted 1: 8 with serum-freemedium (4°C) and added into the upper chamber, and heated at 37°C for 3 h to solidify the gel to each well 10 μL of cell suspension was added to the upper chamber, along with medium containing 10% FBS being added to the lower layer. Cells were cultured for 24 h, cells were fixed then subsequently stained and counted.

### Immunohistochemistry

Thyroid cancer tissue and adjacent tissue samples were fixed in 4% paraformaldehyde, gradient ethanol was used to dehydrate the samples, followed by paraffin-embedding.These tissue samples were subsequently sliced, dewaxed, and hydrated according to conventional procedures, and 3%H_2_O_2_was used to inactivate endogenous peroxides. Tissue samples were incubated with enzymes for 15 min, then a citrate buffer was used for antigen repair. They were further blocked with 10% goat serum at 37°C for 30 min, and incubated with 1: 300 diluted TERT antibody incubated at 4°C overnight. After overnight incubation, the samples were washed in triplicate with PBS buffer, and diluted biotin-labeledII antibody was added and incubated at 37°C for 30 min. Dropwise, streptomycin avidin-peroxidasecomplex working solution was added and further incubated at 37°C for 30 min. Samples were washed three times with PBS and DAB coloring solution was subsequently added. Nuclear stain with hematoxylin was then added, dehydrated with an ethanol gradient, xylene transparent, neutral gum mounted, and dried at 37°C for 48 h. Tissue specimens were observed under a microscope and imaged. The negative controls omitted the antibody and PBS was used instead.

### Statistical analyses

The software GraphPad 8.0 was used to analyze the data. All data were repeated as an independent experiment, in triplicate, and data are expressed as mean ± standard deviation. The significant difference between groups was examined by a two-tailedt-test or one-wayANOVA. (*P< 0.05, **P< 0.01, ***P < 0.001). A *P* < 0.05 was considered significant.

## Results

### Expression ofMiR-195-5p and TERT in thyroid cancer cells and normal cells

**Figure 1.**
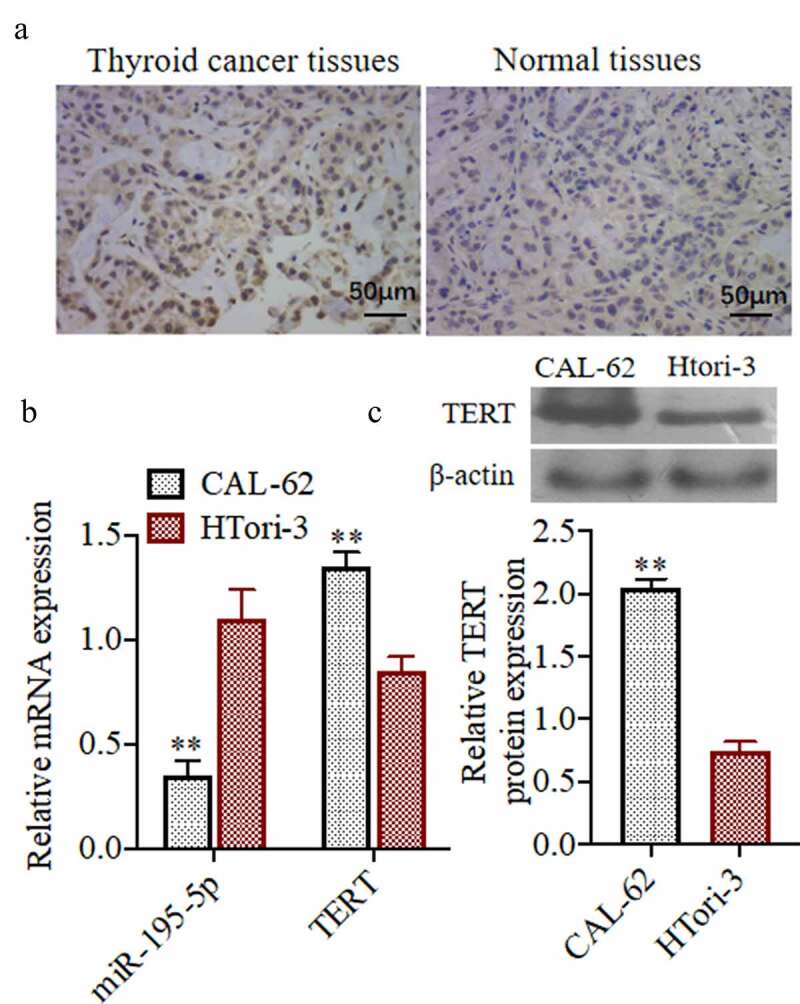
**Expression of miR-195-5p and TERT in thyroid cancer cells and normal cells**. (a)The TERT expression was evaluated by Immunohistochemistry, (200×). (b) The miR-195-5p and TERT mRNA expression was evaluated by qRT-PCR.(c) The TERT protein expression was evaluated by western blot. **P < 0.01 vs. CAL-62 group. Each experiment was repeated three times

To study the roles of miR-195-5p and TERT in thyroid cancer cells and normal cells, we detected the miR-195-5p and TERT expressions by immunohistochemistry, qRT-PCR,and western blot. As shown inFigure 1(a), immunohistochemistry results demonstrated that the level of TERT in human thyroid cancer tissues was higher than that in normal tissues adjacent to the benign lesions. The results of qRT-PCRshowed that miR-195-5p in CAL-62 cells (cancer cells) was lower than in HTori-3 cells (non-cancerouscells), and the expression of TERT was higher in CAL-62 than in HTori-3 cells (Figure 1(b)). According from theFigure 1(c), the results of western blot was also in agreement with the gene expression results in that TERT protein was higher in CAL-62 cells than in HTori-3 cells.

### miR-195-5p overexpression can inhibit the proliferation of thyroid cancer cellCAL-62, induce its apoptosis, and inhibit its invasive ability

**Figure 2.**
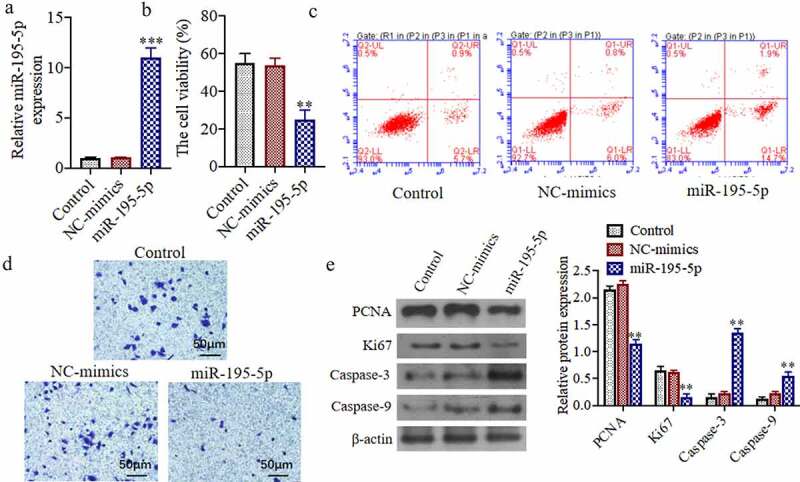
**miR-195-5p overexpression can inhibit the proliferation of thyroid cancer cell CAL-62, induce its apoptosis and inhibit its invasive ability**. (a) The miR-195-5p mRNA expression was evaluated by qRT-PCR.(b) The cell viability was evaluated by MTT assay. (c) The apoptosis of cells was evaluated by flow cytometry. (d) The invasion of cells was evaluated by Transwell, (200×). (e) The Ki67, PCNA, Caspase-3 and Caspase-9 protein expressions were evaluated by western blot. **P < 0.01, ***P < 0.001 vs. Control group

To study the effects of miR-195-5p on the proliferation of thyroid cancer cell, we detected the cell viability, apoptosis, and invasive.Figure 2(a) exhibits the expression of miR-195-5p mimics in the miR-195-5p transfection group was significantly higher than that in the control group and NC-mimicsgroup, demonstrating that the transfection of miR-195-5p was successful in the thyroid carcinoma cells, CAL-62 (P < 0.05). The cell viability of the miR-195-5p mimics group was significantly decreased in comparison to the control group and NC-mimicsgroup (P < 0.05) (Figure 2(b)). Moreover, apoptosis of the miR-195-5p mimics group was significantly increased also (P < 0.05) (Figure 2(c)). Compared to the control group, the number of invasive cells in the miR-195-5p mimics group was significantly reduced (P < 0.05) (Figure 2(d)). As shown inFigure 2(e), the expressions of PCNA and Ki67 protein levels were significantly reduced, while the Caspase-3 and −9 protein expressions were markedly increased in the miR-195-5p mimics group (P < 0.05).

### miR-195-5p can inhibit cell proliferation, induce cell apoptosis, and inhibit invasion ability by knocking down the expression of TERT

**Figure 3.**
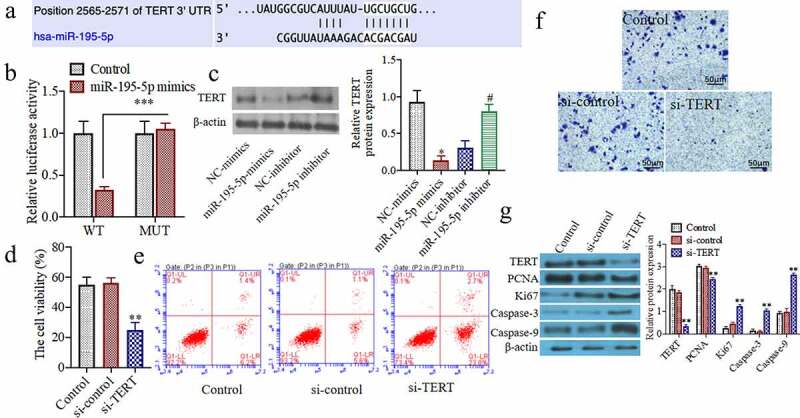
**miR-195-5p targets and regulates the expression of TERT, knocking down the expression of TERT can also inhibit the cell proliferation, induce apoptosis and inhibit invasion ability**. (a) TargetScan predicts miR-195-5p and TERT binding sites. (b) Luciferase activity analysis of the targeted relationship of miR-195-5p and TERT in HEK-293 T cells. (c) The TERT protein expression was evaluated by western blot. (d) The cell viability was evaluated by CCK-8 assay. (e) The apoptosis of cells was evaluated by flow cytometry. (f) The invasion of cells was evaluated by Transwell, (200×). (g) The TERT, Ki67, PCNA, Caspase-3 and Caspase-9 protein expressions were evaluated by western blot. **P < 0.01 vs. Control group or NC mimics,^#^P < 0.05 vs. NC inhibitor

To further study the effects of knocking down the TERT on the proliferation of thyroid cancer cell. As shown inFigure 3(a), the 3ʹ-UTRsequence of TERT contains a nucleotide sequence complementary to miR-195-5p. The results of the dual-luciferasereporter gene detection system showed that the relative activity of dual-luciferaseof WT TERT-WTin the miR-195-5p group was significantly reduced as compared to the miR-congroup (P < 0.05). The relative activity of the dual-luciferaseof the mutant TERT-MUTdid not change significantly (Figure 3(b)). Western blot results showed that the TERT protein expression in the miR-195-5p mimics group was markedly decreased as compared to the NC-mimicsgroup (P < 0.05). Additionally, compared with the NC-inhibitorgroup, protein expression of TERT in the miR-195-5p inhibitor group was significantly increased (P < 0.05) (Figure 3(c)). Moreover, the cell viability in the si-TERTgroup was markedly decreased as compared to the si-congroup (P < 0.05) (Figure 3(d)). The apoptosis of the si-TERTgroup was significantly increased (P < 0.05) (Figure 3(e)). In addition, the number of invasive cells was significantly decreased in the si-TERTgroup compared to the si-controlgroup (P < 0.05) (Figure 3(f)). Furthermore, the TERT, PCNA, and Ki67 protein expressions were significantly reduced, and the Caspase-3 and Caspase-9 protein expressions were markedly increased in the si-TERTgroup (P < 0.05) (Figure 3(g)).

### Overexpression of TERT can partially reverse the effect of overexpression ofMiR-195-5p on cell proliferation, apoptosis, and invasive ability

**Figure 4.**
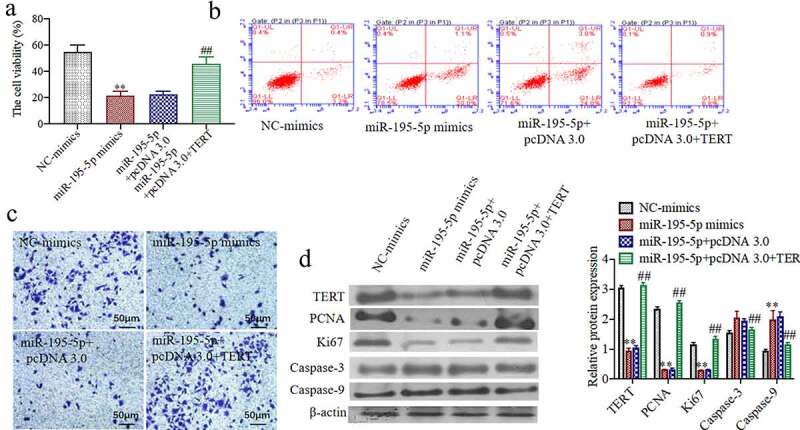
**Overexpression of TERT can partially reverse the effect of overexpression of miR-195-5p on cell proliferation, apoptosis and invasion ability**. (a) The cell viability was evaluated by CCK-8 assay. (b) The apoptosis of cells was evaluated by flow cytometry. (c) The invasion of cells was evaluated by Transwell, (200×). (d) The TERT, Ki67, PCNA, Caspase-3 and Caspase-9 protein expressions were evaluated by western blot. **P < 0.01 vs. NC-mimicsgroup,^##^P < 0.01 vs. miR-195-5p + pcDNA 3.0 group

To study whether on the overexpression of TERT can partially reverse the effect of overexpression of miR-195-5p on cell proliferation, apoptosis, and invasive ability. In comparison to the miR-195-5p mimics + pcDNA 3.0 group, the cell viability of CAL-62 cells was significantly increased in the miR-195-5p mimics + pcDNA 3.0 + TERT group (P < 0.05) (Figure 4(a)). The apoptotic cells were markedly decreased in the miR-195-5p mimics + pcDNA 3.0 + TERT group (P < 0.05) (Figure 4(b)). Moreover, the number of invasive cells was significantly increased in the miR-195-5p mimics + pcDNA 3.0 + TERT group (P < 0.05) (Figure 4(c)). The western blot results showed that the TERT, PCNA, and Ki67 protein expressions were significantly increased, while the expressions of Caspase-3 and Caspase-9 were markedly reduced in the miR-195-5p mimics + pcDNA 3.0 + TERT group (P < 0.05) (Figure 4(d)). These results indicate that overexpression of TERT can partially reverse the proliferation of thyroid cancer cells, CAL-62, through the overexpression of miR-195-5p and induce its apoptosis and inhibit its invasive ability.

## Discussion

With the continuous in-depthresearch of molecular biology, it has been shown that lncRNAs play an important role in the growth and development of individuals, cell proliferation, apoptosis, and the occurrence and development of tumor diseases [20]. TERT is related to the proliferation, invasion, and migration of various malignant tumor cells [21]. For example, studies have demonstrated that TERT is highly expressed in gastric cancer tissues and is related to lymph node metastasis [22]. miRNA is a single-strandednon-codingsmall RNA that is abnormally expressed in many diseases [23,24]. In particular, miRNA participates in biological metabolic processes as either in an oncogene or a tumor suppressor gene capacity and is closely related to tumor occurrence. miR-195-5p is one of the important members of the miR-15/16/195/424/497 family [25]. Low expression of miR-195-5p has been confirmed in a variety of tumors and may be associated with tumorigenesis and development [26,27]. Shao et. al., demonstrated that miR-195-5p is downregulated in cervical cancer serum and tissue samples, and can increase cancer cell invasiveness by regulating the target gene cyclin D1, which can be used as its therapeutic target [28]. In our current study, we also demonstrate that miR-195-5p is lowly expressed in thyroid cancer tissues and cells, and TERT mRNA and protein are highly expressed.

PCNA is an important protein that initiates cell proliferation and differentiation [29]. The higher the PNCA index, the faster the cell division and proliferation will occur, which promotes the ability of cells to proliferate indefinitely and change the morphological structure and function of the cell [30]. Proliferating cell cycle-associatednuclear antigen (Ki67) is a protein antigen related to cell division and proliferation in the nucleus. The expression of Ki67 is closely related to cell proliferation, is a sensitive indicator reflecting cell proliferation, and is important for regulating the cell cycle [31]. Ki67 plays an important role in maintaining structure [32]. The present study showed that overexpression of miR-195-5p inhibits TERT, which significantly reduces the presence of Ki67 and PCNA in thyroid cancer cells CAL-62, and inhibits the proliferation of CAL-62. Thereby effectively alleviating the symptoms in thyroid cancer patients.

Apoptosis is a programmed cell death regulated by genes in which Caspase is essential for this process. Caspase-3 and Caspase-9 are apoptotic factors of the Caspase family [33]. Caspase-9 belongs to the apoptotic mover and is the key protease of the mitochondrial apoptotic pathway, at the start of the activation process; Caspase-3 is the main executor of apoptosis. Caspase-9 is activated through certain processes, whereby it is subsequently cleaved to activate proCaspase-3. Active Caspase-3 can cleave other Caspase substrates causing a cascade reaction and eventually targeting cell apoptosis [34]. In this study, the expression of Caspase-3 and Caspase-9 in miR- 195 mimic + TERT group cells was significantly increased, which may indicate that over-expressedmiR-195-5p mimics effectively increased apoptotic factors, causing thyroid cancer cells to be rapidly inhibited. Some studies have also found that the increase of miR-195-5p can promote the proliferation and migration of cancer cells. We speculate that the different functions of miR-195-5p in different tumor cells may be due to the level of miR-195-5p expression, which is pertinent for further evaluation.

## Conclusion

In summary, overexpression of miR-195-5p inhibits the regulatory effect of TERT on the proliferation, apoptosis, and movement of thyroid cancer cells, CAL-62. The mechanism may be through the reduction of Ki67 and PCNA levels and inhibition of proliferation of cancer cells; The Caspase-3 and Caspase-9 promote the apoptosis of cancer cells; thereby inhibiting the ability of cancer cell invasion.

## Data Availability

All data are available from the corresponding authors on reasonable request.
